# Mobile Technology Application for Improved Urine Concentration Measurement Pilot Study

**DOI:** 10.3389/fped.2018.00160

**Published:** 2018-06-06

**Authors:** Laura Walawender, Jeremy Patterson, Robert Strouse, John Ketz, Vijay Saxena, Emily Alexy, Andrew Schwaderer

**Affiliations:** ^1^Pediatric Residency, Nationwide Children's Hospital, Columbus, OH, United States; ^2^User Experience Technology Research and Development, Nationwide Children's Hospital, Columbus, OH, United States; ^3^Center for Clinical and Translational Medicine, Nationwide Children's Hospital, Columbus, OH, United States; ^4^Division of Nephrology, Department of Pediatrics, Indiana University, Indianapolis, IN, United States; ^5^Biostatistics Core, Nationwide Children's Hospital, Columbus, OH, United States

**Keywords:** thirst, cell phone apps, hydration, specific gravity, urine colors

## Abstract

**Objectives:** Low hydration has a deleterious effect on many conditions. In the absence of a urine concentrating defect, urine concentration is a marker of hydration status. However, markers to evaluate hydration status have not been well studied in children. The objectives of this paper are to compare measures of thirst and urine concentration in children and to develop a novel mobile technology application to measure urine concentration.

**Study Design:** Children age 12–17 years were selected (*n* = 21) for this pilot study. Thirst perception, specific gravity (automated dipstick analysis and refractometer), and urine color scale results were correlated to urine osmolality. The technology department developed a mobile technology camera application to measure light penetrance into urine which was tested on 25 random anonymized urine samples.

**Results:** The patients' thirst perception and color scale as well as two researchers color scale did not significantly correlate with osmolality. Correlation between osmolality and hydration markers resulted in the following Pearson coefficients: SG automated dipstick, 0.61 (*P* 0.003); SG refractometer, 0.98 (*P* < 0.0001); urine color scale (patient), 0.37 (*P* 0.10), and light penetrance, −0.77 (*P* < 0.0001). The correlation of light penetrance with osmolality was stronger than all measures except SG by refractometer and osmolality.

**Conclusion:** The mobile technology application may be a more accurate tool for urine concentration measurement than specific gravity by automated dipstick, subjective thirst, and urine color scale, but lags behind specific gravity measured by refractometer. The mobile technology application is a step toward patient oriented hydration strategies.

## Background

Hydration is an important component of health. Excessive dehydration, defined as loss of ≥5% of body weight has long been associated clinical sequalae including decreased peripheral perfusion, low pH and high urea necessitating oral or intravenous fluid resuscitation (1). Escalating evidence indicates that even mild dehydration, known as hypohydration, may be a disease modifying factor for multiple pediatric public health concerns. The prevalence of hypohydration, defined as a urine osmolality ≥800 milliosmoles, is 54.5% in United States children (2). Pediatric hypohydration is associated with a range of conditions including functional constipation, headaches and obesity (3–5). Further, hypohydration is associated with decreased cognitive performance, with cognition improving as urine osmolality decreases (6, 7) Maintenance of hydration is also an important factor for pediatric subspecialties. For instance, in nephrology increased hydration may reduce urinary stone recurrence and cyst progression in ADPKD (8–10).

Obtaining adequate hydration may be particularly challenging in pediatrics because children have higher surface areas relative to mass, are more dependent on caregivers for fluid intake, and have different thirst sensitivities compared to adults (11). A study of soccer players aged 8–15 years examined urine specific gravity and showed that before starting practice for the day, two-thirds of the children were significantly dehydrated and they were not able to achieve a healthy hydration level by the end of practice (12). Additionally, children do not change their hydration practice based upon how they perceive their hydration (13). Therefore, strategies to maintain hydration in children represent a clinically important research gap.

Children generally have a high surface area to body mass ratio and an underdeveloped sweating mechanism which makes them less effective at thermoregulation compared to adults. Hydration is important for maintaining thermoregulatory capacity and hydration depends on thirst. Children may become dehydrated, even when allowed to *ad lib* drink during exercise (14). The prevalence of inadequate hydration, defined as urine osmolality >800 mOsm/L, is 55% among United States children (2).

A range of methodologies have been used to assess hydration including plasma osmolality, thirst perception measures and measures of urine concentration such as urine osmolality, urine specific gravity and urine color scale (13, 15, 16). Urine concentration is a marker of hydration status and urine osmolality >800 mOsm/kg has been used as a “gold standard” to represent dehydration, however use of osmolality is generally not available at the point of care (13, 17). Patient-centered methodologies represent an emerging strategy to increase patient engagement in treatment plans and adherence. Therefore, expanding measures of urine concentration/patient hydration status to include a mobile technology platform represents a step toward a patient centered strategy to improve clinical outcomes (18).

The objective of this study is to assess pediatric patients' reported level of thirst, urine specific gravity and urine color compared to urine osmolality. This study is important because it compares a new tool for assessing hydration/urine concentration against these current methods. Specifically, this study developed and tested a new mobile technology application that measures urine light penetrance and estimates osmolality and compared it to other measures of hydration as a point of care tool to inform users of hydration status.

## Methods

### Enrollment of children

This pilot study was approved by the Nationwide Children's Hospital (NCH) Institutional Review Board (IRB16-00815). Children age 12–17 years were eligible for enrollment. They were voluntarily enrolled from Nephrology and Urology clinics during already scheduled appointments and written consent and assent were obtained. Exclusion criteria were chosen to prevent urine samples from being falsely dilute or having altered color. Children were excluded if they had chronic kidney disease greater than stage 1, a known concentrating defect, diuretic use, gross hematuria, or were on a medication known to change urine color (19).

### Urine concentration/hydration tests performed by patients

(a) Visual analog scale: This tool consists of a line (14.5 cm in our case) with the following format:


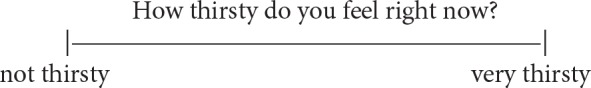


The patients objectify their thirst by placing a mark that is measured by research staff as distance from the beginning left side in mm. The validation and use of this method has been previously described (15). (b) Urine color scale*:* The patients picked the urine color that best matched their urine sample using an 8 point urine color scale (http://www.ncaa.org/sites/default/files/Assess+Your+Hydration+Status.pdf).

### Urine concentration tests performed by research staff

Urine specific gravity (SG) was measured by Clinitek automated dipstick analysis (Siemens, Malvern PA, USA) and refractometer (Master-URC/NM, Atago, Tokyo Japan); urine osmolality was measured by osmometer (Advanced Instruments, Norwood MA, USA); urine samples were matched to the urine color scale by four research team members independently. Research team members are referred to as RT1, RT2, RT3, and RT4.

### Development of mobile technology application

The Nationwide Children's Technology Department developed a cell phone camera application to measure light penetrance into urine. The device software is a custom iOS-based native binary running on a 6th generation Apple iPod Touch, developed in a combination of Objective-C, Objective-C++, and C++. The application binary utilizes optical sensors, or specifically the device's back-facing camera, to capture a series of images from the specimen receptacle. The capture software guides the operator through the image capture process via visual tools and feedback that assist the operator in the process of positioning the specimen container in the appropriate location best for optimal image captures. To standardize for ambient light, a standardization adaptor was made to fit a 100 ml high density polyethylene urine collection cup and printed with a 3D printer.

After the urine collection container is placed the standardization adaptor, the operator initiates the image capture sequence via the software interface. The application activates the device's back-facing flash and additionally locks the camera focal length, exposure, and white balance level to ensure consistent image capture across samples. The software will then commence capture of four full-frame images from the illuminated sample receptacle.

Once the full-frame image capture completes, the software derives the mean perceived luminance value using the standard luminance formula. The software begins the analysis by extracting and storing two 760 × 132 dimension rectangles from each of the four captured images (**Figure 2A**). Each image is then downsampled by a factor of 5, resulting in 8 stored images at 152 × 26 resolution. Downsampling is employed to increase analysis performance and does not significantly change the resulting mean perceived luminance value of the sample.

The application calculates mean perceived luminance with the 3,952 RGB values contained in the eight captured image samples. Luminance is derived per pixel via the formula:

$$\begin{array}{l} \sqrt{0.299{\left(\frac{R}{255}\right)}^{2}+0.587{\left(\frac{G}{255}\right)}^{2}+0.144{\left(\frac{B}{255}\right)}^{2}} \end{array}$$

Where R = Red channel value in RGB color space with scale 0–255, G = Green channel value in RGB color space with scale 0–255 and B = Blue channel value in RGB color space with scale 0–255. The 8 subsampled mean perceived luminance values are summed and averaged for one total mean average luminance value, used as an indication of the sample's specific density.

### Collection of anonymized urine samples

Random urine samples without gross hematuria were collected to test the mobile technology application using NCH IRB0 00383. These samples were obtained because ≥50 ml of urine was needed to fill the urine collection container enough that the image obtained by camera was entirely fluid when using the mobile technology application and this volume was not collected on most consented patients.

### Tests performed on anonymized urine samples

Light penetrance using the mobile technology application and osmolality were measured on all random anonymized samples. A subset of anonymized urine samples were randomly selected for repeated measurements to measure test-retest reliability of the mobile technology application.

### Statistical analysis

Linear regression compared urine osmolality to the other hydration markers including specific gravity by dipstick, specific gravity by refractometer, urine color on the urine scale as determined by the patient and the research group members, thirst, and light penetrance.

Correlations with osmolality were run for all measures of urine concentration. The distribution of each measurement was checked for normality—most variables deviated from normality, so Spearman correlations were run in addition to Pearson correlations; Spearman and Pearson correlation coefficients and corresponding *p*-values are reported for each measure; *p*-values < 0.05 indicate that the correlation is significantly different from 0.

The strength of the correlation of light penetrance with osmolality was compared to the strength of the correlations of other measurements with osmolality using the test of difference between two independent correlations. Absolute values of correlation coefficients were used for these calculations (negative signs were ignored). *P*-values indicating whether there is a significant difference between the two correlations were obtained.

Finally, the reliability of the light penetrance application was assessed using Cronbach's standardized alpha on the three repeat application measurements.

GraphPad Prism (La Jolla, Ca) was used to perform Linear Regression. SAS version 9.4 (Cary, NC) and R version 3.1.1 were used for all other analyses.

## Results

### Patient enrollment

A total of 21 patients were enrolled. There were 9 males (43%) and 12 females (57%). The average age of the patients was 14.95 ± 1.69 (range 12–17) years old. Eighteen patients were seen in nephrology clinic (85.7%), 2 in urology clinic (9.5%), and 1 in combined Urology/Nephrology clinic (4.8%). Eight patients were being seen for blood pressure or hypertension (38.1%), 3 for kidney stones and hypercalciuria (14.3%), 2 for hematuria (9.5%), 1 for polycystic kidney disease (4.8%), and the remaining 7 (33.3%) were seen for other reasons.

### Evaluation of historical urine concentration and hydration measures

**Figure 1:**
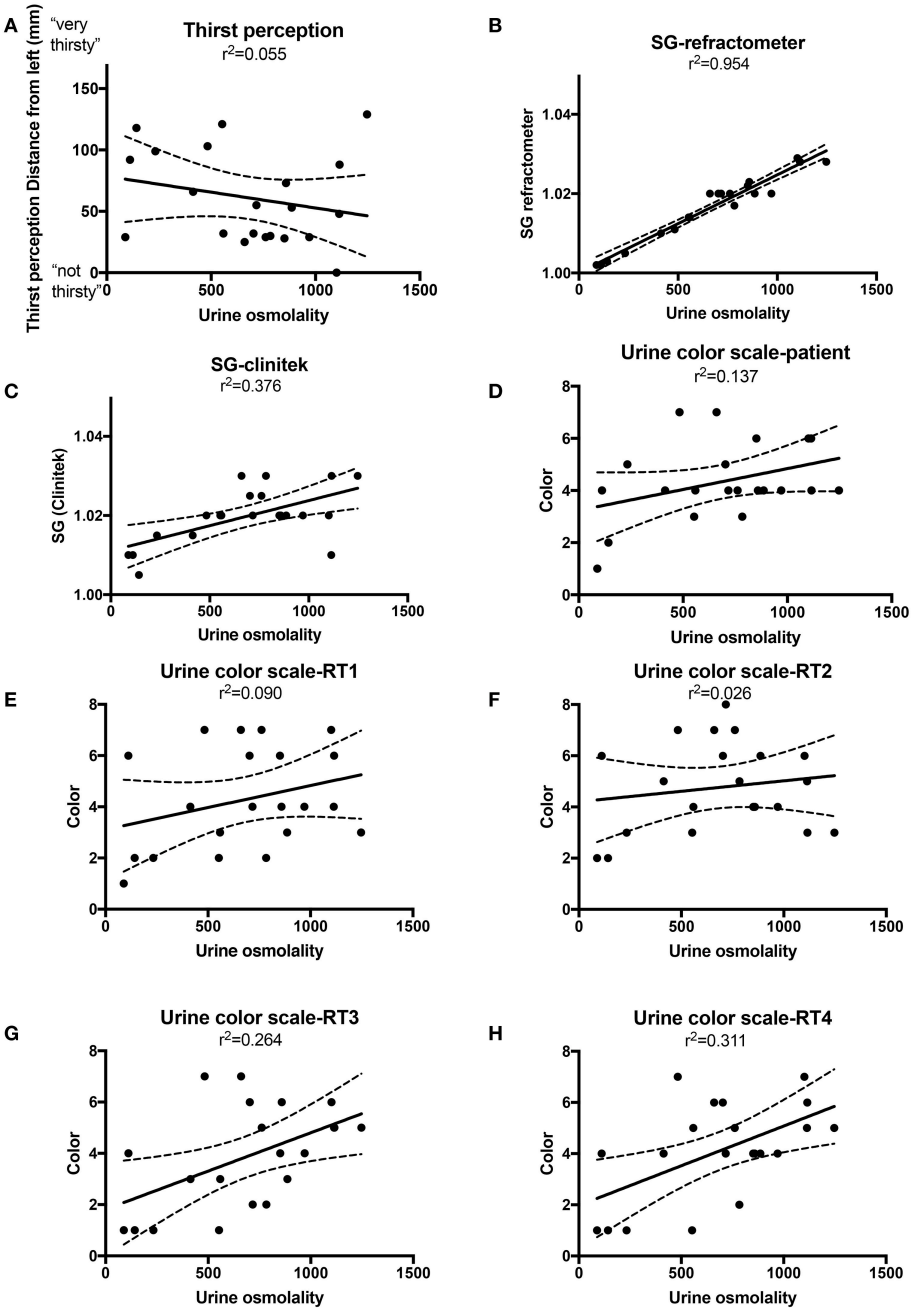
Linear regression with best fit (solid line) and 95% confidence intervals (dashed lines) for urine osmolality vs. thirst perception **(A)**, specific gravity (SG) by refractometer **(B)** SG by Clinitek automated urine dipstick reader **(C)**, Urine color scale patient **(D)**, Urine color scale research team (RT) members 1–4 **(E–H)**.

Linear regression comparing osmolality to hydration markers resulted in the following R squared values: specific gravity by automated dipstick, 0.376; specific gravity by refractometer, 0.954; urine color scale (patient), 0.137; urine color scale (research group), 0.026–0.311; and thirst, 0.055. (Figures 1A–H). The slopes of the SG automated dipstick and SG refractometer were significantly deviated from a zero-slope line when compared to urine osmolality.

Specific gravity (as measured by a refractometer or Clinitek), and urine color scale (as measured by RT1 or RT2) were significantly correlated with osmolality. Urine color scale (as measured by patient, RT3, or RT4) and thirst perception did not have correlations with osmolality that differed significantly from zero (Table 1). Spearman results were similar to Pearson results and did not change conclusions.

**Table 1:** Correlations between hydration markers and urine osmolality.

**Measurement**	***N***	**Pearson coefficient**	***p*-value**	**Spearman coefficient**	***p*-value**
Thirst perception	21	−0.24	0.30	−0.20	0.40
SG: refractometer	21	0.98	^*^<**0.0001**	0.94	^*^<**0.0001**
SG: Clinitek	21	0.61	^*^0.003	0.55	^*^0.01
Color scale: Pt	21	0.37	0.10	0.25	0.26
Color scale: RT1	21	0.56	^*^0.01	0.46	^*^0.04
Color scale: RT2	21	0.51	^*^0.02	0.46	^*^0.04
Color scale: RT3	21	0.30	0.19	0.26	0.25
Color scale: RT4	21	0.16	0.49	0.08	0.74

* *Statistically significant*.

*Bold values are statistically significant*.

### Collection of anonymized urine samples

Thirty-one anonymized urine samples were collected and 25 contained sufficient urine volume needed for the mobile technology application. Fifteen of these samples were randomly selected to measure test-retest reliability of the application.

### Development and testing of the mobile technology application

**Figure 2:**
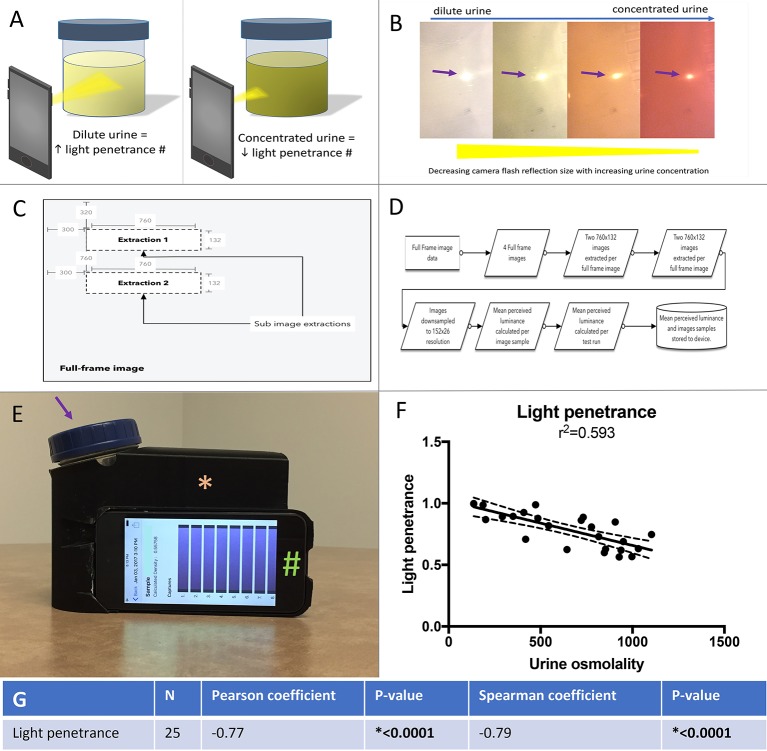
**(A)** Left panel, a dilute urine sample results in in increased light penetrance and increased diameter of camera flash. The camera flash is being used to illustrate light penetrance, but was not directly measured for the mobile technology application. Right panel, a concentrated urine sample results in decreased light penetrance and decreased diameter of camera flash. **(B)** The correlation between urine concentration, camera flash diameter (arrow) and light penetrance is demonstrated. **(C)** Full-frame with sub-image extraction. **(D)** Capture and analysis workflow. **(E)** An example of the urine collection container (arrow) and ipod (#) in the 3D printed adaptor (asterisk), (to standardize for variable room light levels and distance from camera to urine sample). **(F)** Linear regression with best fit (solid line) and 95% confidence intervals (dashed lines) for urine osmolality vs. light penetrance as measured by the mobile technology application. **(G)** Spearman and Pearson's correlations and associated p values for correlation between light penetrance and urine osmolality.

We developed a mobile technology application based on the premise that light penetrance is inversely proportional to urine osmolality. (Figures 2A,B). The application full-frame with sub-image extraction along with capture and analysis workflow are depicted in Figures 2C,D. Example of the 3D printed standardization chamber (to make light exposure consistent), urine collection container and iPod with running application are presented in Figure 2E. Using linear regression, the *R*^2^ between and light penetrance and urine osmolality was 0.593 (Figure 2F). There was a significant negative correlation between light penetrance and osmolality (Figure 2G). The test-retest reliability of the application was tested. The standardized Cronbach's alpha for three measurements of 15 randomly selected samples taken with the light penetrance app was 0.997, indicating excellent retest reliability.

### Comparison of urine concentration measures

The correlation between osmolality and light penetrance (as measured by the application) is significantly stronger than the correlations between osmolality and urine color scale—patient, urine color scale—RT3, thirst perception, and urine color scale—RT4. The correlation between osmolality and light penetrance is significantly weaker than the correlation between osmolality and specific gravity—refractometer. The correlations between osmolality and specific gravity—Clinitek, urine color scale—RT1, and urine color scale—RT2 did not differ significantly from the correlation between osmolality and light penetrance (Table 2).

**Table 2:** Strength of correlation between light penetrance and osmolality compared to correlation between other hydration markers and urine osmolality.

**Measurement**	**Pearson correlation comparison *p*-value**	**Spearman correlation comparison *p*-value**	**Stronger or weaker association than light penetrance**
SG: refractometer	^*^<0.0001	^*^0.04	Stronger
SG: Clintek	0.33	0.15	Nonsignificant trend weaker
Color scale: Pt	^*^0.05^∧^	^*^0.01	Weaker
Color scale: RT1	0.22	0.07	Nonsignificant trend weaker
Color scale: RT2	0.15	0.07	Nonsignificant trend weaker
Color scale: RT3	^*^0.03	^*^0.01	Weaker
Color scale: RT4	^*^0.01	^*^0.002	Weaker
Thirst perception	^*^0.01	^*^0.01	Weaker

* *Statistically significant*.

∧ *p-value = 0.0466, therefore assigned significance even though it rounds to 0.05*.

*Bold values are statistically significant*.

The raw data obtained for this experiment and used for statistics is presented in Supplemental File 1.

## Discussion

This study evaluated different markers to help identify potential point of care tests to evaluate urine concentration as an estimation of hydration status. We used urine osmolality as the gold standard and compared urine specific gravity measured by refractometer, urine specific gravity measured by automated dipstick reader, urine color as determined by urine color scale and thirst perception as determined by visual analog scale. Last, we developed a mobile device application that used the camera and camera flash to estimate urine concentration by light penetration.

Thirst perception is heavily relied upon as a means of assessing and maintaining adequate hydration. Unfortunately, an individual needs to have lost enough of their total body water to have a 1–2% decrease in body mass before thirst becomes apparent (20). A study of athletes that dehydrate to lose weight showed that thirst perception scale was only able to identify extreme dehydration but not mild dehydration (21). The results of our study showed that thirst did not correlate with urine osmolality. This suggests that thirst is not an adequate marker of hydration or a useful tool for maintaining hydration. Moreover, newer research suggests that thirst may actually work through feedforward regulation rather than as a negative feedback loop to maintain homeostasis further supporting that thirst should not be used a marker of hydration status (22). Our findings indicate that perception varies between children and does not appear to be a reliable determinant of hydration status. Whether altered thirst perception segregates to patients with distinct phenotypes such as kidney stones remains to be seen.

In clinical practice, urine specific gravity is the most widely used estimation of urine concentration. Urine specific gravity measured by refractometer but not Clinitek was strongly correlated with urine osmolality. The *R*^2^ for the correlation between urine specific gravity and urine osmolality was 0.9 by refractometer and 0.6 by Clinitek automated dipstick. The poor reliability of urine dipstick specific gravity and good reliability with urine refractometer specific gravity is consistent with what has been previously reported. A study by Roessingh and colleagues reported an *R*^2^ of 0.9 by refractometer, 0.6 by Clinitek automated urine dipstick and 0.5–0.6 by visual readings of urine dipstick (23) While urine specific gravity measured by refractometer is an accurate estimation of urine osmolality, it would likely not be practical for patients to use from day to day.

Armstrong and colleagues developed an 8-point color scale and reported a linear relationship between urine color and both urine specific gravity along with urine osmolality (24) In prior studies correlating urine osmolality to an 8-point color scale, the *R*^2^ for the correlation was 0.45 when studied in healthy Greek children and between 0.31 and 0.71 when studied in nursing home residents (13, 25). We had similar results to the aforementioned study with an *R*^2^ of 0.37 when the patients matched the colors and between 0.16 and 0.56 when research team members matched the colors. Although the 8-point urine color scale is convenient to use and readily available, it does not appear to be particularly reliable or yield consistent results between users.

Mobile technology/smartphone use is an emerging strategy in care innovation research. Randomized controlled trials have demonstrated improved adherence to a range of interventions including diabetes plans resulting in decreased hemoglobin A1c, weight loss plans and dietary recording (26, 27). Because of the success of mobile technology in improving adherence in other conditions and lack of current strategies to increase fluid intake we have begun to develop a mobile technology platform to incorporate into fluid management. This strategy might be applicable to use from day to day in adolescents.

## Limitations

This study had limitations. The relatively limited number of patients decreases the power of the study. Future studies with a larger sample size will be needed to perform subgroup analysis to evaluate the effects of sex, age, and race/ethnicity. Sampling patients from the nephrology and urology clinics makes the study less applicable to the general pediatric population; however, patients from the population sampled are more likely to have conditions requiring active monitoring of hydration status. The volume of urine required for the cell phone application to work, ≥50 ml, prevented analysis of the samples provided from the consented clinic patients in this case in which a smaller aliquot of urine was acquired prior to application development. While 50 ml volume is feasible to collect from school age children, it might be problematic in younger pediatric patients (28). However, we speculate that the *R*^2^ for correlation between light penetrance and urine osmolality may have been higher in the clinic patients since the consented clinic patient samples were screened for more covariates than the random clinic samples. Future studies could investigate whether screening urine samples with a urinalysis for factors such as presence of blood or bilirubin may improve the accuracy of the mobile technology application. The 50 ml volume requirement along with the requirement for a standardization chamber would be barriers for application use in clinical practice and would need to be addressed to optimize this modality for everyday use. Modifications to require lower urine volume requirements and/or alleviate the need for a standardization chamber will make this technology more practical for patients.

## Conclusions

In children, thirst perception did not correlate with urine concentration highlighting the need for new technology to monitor hydration status. Urine osmolality and urine specific gravity by refractometer were the most accurate measures of urine concentration as a measure of hydration status. Both methods require costly equipment and special training to use. By linear regression correlation with urine osmolality, our mobile technology application had a *R*^2^ of 0.593, while the methodology used most frequently in our day to day care of patients, S.G. by automated dipstick, had a *R*^2^ of 0.376. We submit that it represent movement toward improved accuracy of urine concentration/hydration measurement that can be adapted by use by patients. Improving the *R*^2^ of the mobile technology application closer to the *R*^2^ attainable by SG measured by refractometer and urine osmolality and ensuring that results can be duplicated by patients will be key steps in implementing our devise into clinical care. There are many health conditions that would greatly benefit from better point of care hydration assessment including chronic constipation, obesity, headaches, urinary stones, and autosomal recessive polycystic kidney disease. None of the current point of care methods that were evaluated (urine color scale, urine specific gravity by automated dipstick, and thirst) appeared to be ideal tools for addressing hydration. Our novel mobile technology application correlated the best with urine osmolality and is a promising new tool that could be modified for point of care assessment of urine concentration and hydration status by both healthcare providers and patients.

Future directions of this research include expanding the application. It currently only reports the light penetrance of the sample. Additional programing could allow the application to incorporate a hydration goal that could be personalized to each user and then report, based upon the light penetrance, if the user is well hydrated or poorly hydrated. The application would then need to be formally tested when used by children and adolescents.

## Disclosures

AS has received financial compensation from Allena Pharmaceuticals on a topic unrelated to the content of this manuscript.

## Ethics statement

This study was carried out in accordance with the recommendations of Nationwide Children's Hospital Institutional Review Board (IRB). The protocol was approved by the Nationwide Children's Hospital IRB. All subjects gave written informed consent in accordance with the Declaration of Helsinki. Children also gave written assent. A waiver of consent from Nationwide Children's Hospital IRB was obtained for collection of random anonymized urine sample.

## Author contributions

LW completed the 1st draft of the manuscript. All authors critically reviewed and edited the manuscript. Investigation was completed by LW, JK, VS, and AS. Formal analysis was completed by AS, LW, JP, RS, and EA. Conceptualization/design and Methodology were completed by ALS, LW, JP, and RS.

### Conflict of interest statement

The authors declare that the research was conducted in the absence of any commercial or financial relationships that could be construed as a potential conflict of interest.
